# Neonatal Brain Injury and Genetic Causes of Adult-Onset Neurodegenerative Disease in Mice Interact With Effects on Acute and Late Outcomes

**DOI:** 10.3389/fneur.2019.00635

**Published:** 2019-06-18

**Authors:** Lee J. Martin, Margaret Wong, Allison Hanaford

**Affiliations:** ^1^Division of Neuropathology, Department of Pathology, Johns Hopkins University School of Medicine, Baltimore, MD, United States; ^2^Department of Neuroscience, Johns Hopkins University School of Medicine, Baltimore, MD, United States; ^3^Pathobiology Graduate Training Program, Johns Hopkins University School of Medicine, Baltimore, MD, United States

**Keywords:** neonatal brain damage, traumatic brain injury, ALS, synuclein oligomerization, Parkinson's disease

## Abstract

Neonatal brain damage and age-related neurodegenerative disease share many common mechanisms of injury involving mitochondriopathy, oxidative stress, excitotoxicity, inflammation, and neuronal cell death. We hypothesized that genes causing adult-onset neurodegeneration can influence acute outcome after CNS injury at immaturity and on the subsequent development of chronic disability after early-life brain injury. In two different transgenic (Tg) mouse models of adult-onset neurodegenerative disease, a human A53T-α-synuclein (hαSyn) model of Parkinson's disease (PD) and a human G93A-superoxide dismutase-1(hSOD1) model of amyotrophic lateral sclerosis (ALS), mortality and survivor morbidity were significantly greater than non-Tg mice and a Tg mouse model of Alzheimer's disease after neonatal traumatic brain injury (TBI). Acutely after brain injury, hαSyn neonatal mice showed a marked enhancement of protein oxidative damage in forebrain, brain regional mitochondrial oxidative metabolism, and mitochondriopathy. Extreme protein oxidative damage was also observed in neonatal mutant SOD1 mice after TBI. At 1 month of age, neuropathology in forebrain, midbrain, and brainstem of hαSyn mice with neonatal TBI was greater compared to sham hαSyn mice. Surviving hαSyn mice with TBI showed increased hαSyn aggregation and nitration and developed adult-onset disease months sooner and died earlier than non-injured hαSyn mice. Surviving hSOD1 mice with TBI also developed adult-onset disease and died sooner than non-injured hSOD1 mice. We conclude that mutant genes causing PD and ALS in humans have significant impact on mortality and morbidity after early-life brain injury and on age-related disease onset and proteinopathy in mice. This study provides novel insight into genetic determinants of poor outcomes after acute injury to the neonatal brain and how early-life brain injury can influence adult-onset neurodegenerative disease during aging.

## Introduction

Epidemiological and molecular genetics studies have identified definitive genetic, environmental, and lifestyle risk factors for age-related neurodegenerative diseases (1–3). Most forms of Alzheimer's disease (AD), Parkinson's disease (PD), and amyotrophic lateral sclerosis (ALS) are sporadic (idiopathic) with no known inheritance pattern or genetic associations and are respectively the 1st, 2nd, and 3rd most common adult-onset neurodegenerative diseases (1, 4). Some familial forms of AD, PD, and ALS link to gene mutations (1, 5, 6). Along the lifelong spectrum, individuals can suffer from brain and spinal cord traumatic and ischemic injuries. Traumatic brain injury (TBI) at all ages is an enormous public health concern that embraces sports-, vehicular crash-, and warfare-related incidents, events linked to neurobehavioral, cognitive and motor deficits (7). About 5.3 million people in the US are living with some form of TBI-related disability (7). There is a connection between acute brain injury and chronic age-related neurodegenerative disease. For example, a history of head trauma is associated with AD (8), PD (9–11), and ALS (12, 13). A recent study of 7,130 participants identified a strong association of TBI with risk of PD in late-life (9). A greater than expected prevalence of ALS is found in Italian professional soccer players (13). Studies confirmed that risk for ALS is higher than expected in professional soccer players and extend the result to university athletes (14, 15). Other studies suggest associations between chronic traumatic encephalopathy in US athletes and motor neuron disease (12) and associations between TBI and ALS in the general population (16, 17). Importantly, TBI can promote proteinopathies involving human transactive DNA-binding protein 43 (hTDP43) and α-synuclein (hαSyn) (18). However, generally, these associations are for mature brain neuropathology. It is not known whether neonatal, pediatric, or juvenile CNS injury affects the aging brain and age-related neurodegenerative diseases and, inversely, if genetic variations linked to age-related neurodegenerative disease influence outcomes from acute CNS injury in early life. Poor outcome after hypoxia-ischemia in piglet newborns is associated with a variety of physiological abnormalities during early recovery (19), but the possibility of underlying genetic risk factors that might drive poor outcomes in acute injury settings in infants and children is unclear. Emerging data support this possibility. Single nucleotide polymorphisms in genes related to dopamine neurotransmission have roles in short- and long-term neurobehavioral recovery after early childhood TBI (20). *Catalase* gene (21) and *interleukin-6* gene (22–24) polymorphisms are associated with higher susceptibility to cerebral palsy after neonatal hypoxia-ischemia. Glial glutamate transporter gene *EAAT2* polymorphisms associate with cerebral palsy in preterm infants (25). Indeed, mitochondriopathy, oxidative stress, excitotoxicity, intracellular calcium stress, inflammation, and neuronal cell death are all putative mechanisms in neonatal brain damage (4, 26) and age-related neurodegenerative disease (4). We hypothesized that the genetics of AD, PD, and ALS can influence acute outcome after brain injury at immaturity and the subsequent development of adult-onset chronic neurodegenerative disease by aggravating proteinopathy.

## Materials and Methods

### Mice

We used transgenic (Tg) mice expressing human mutant A53T-α-synuclein (hαSyn) (27), human mutant G93A-superoxide dismutase-1(hSOD1) (28), and human mutant amyloid precursor protein-presenilin 1 (hAPP-PS1) (29). Colonies of these mouse lines and their genotyping have been used by us before (27, 30–32). Non-transgenic (non-Tg) littermates were controls. The institutional Animal Care and Use Committee approved the animal protocols. Because each mutant mouse line has its own background strain, non-trangenic controls were strain specific for each mutant line.

### Unilateral Closed Skull Cortical Contusion Injury Model (CCI) in Neonatal Mice

**Figure 1:**
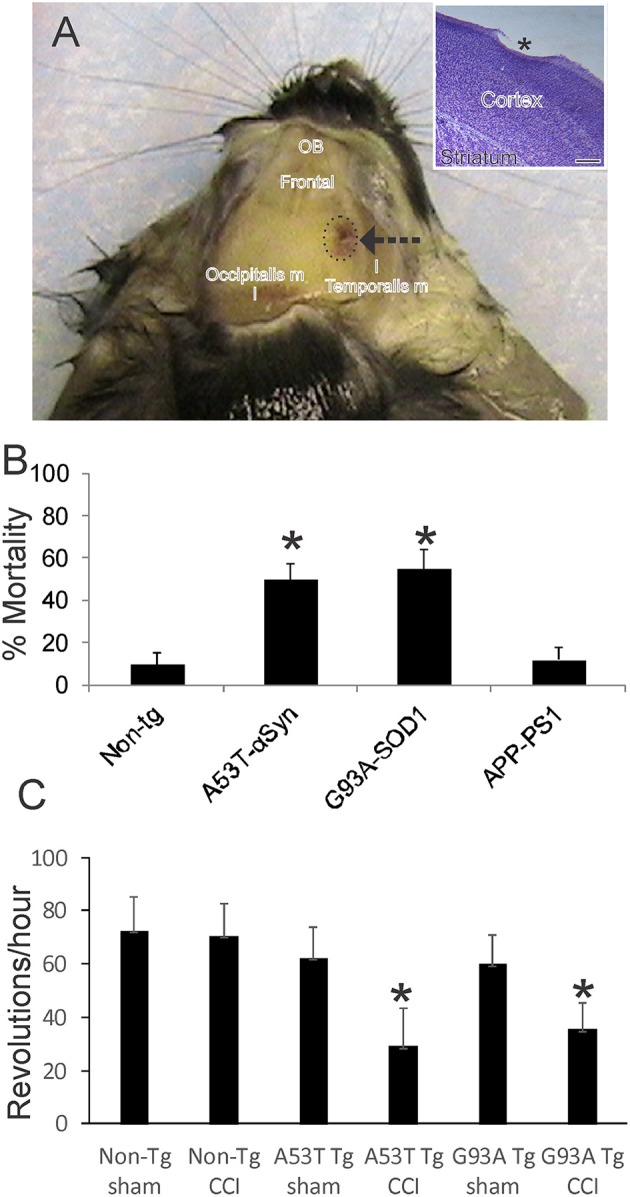
Mouse model of mild closed skull cortical contusion injury (CCI) and clinical outcomes. **(A)** Head of a postnatal day 30 mouse that received a right side CCI (arrow) on postnatal day 7. Surrounding skull muscles and some underlying brain structures, olfactory bulbs (OB) and frontal cortex (frontal), are identified for orientation. Inset shows mild injury to frontal cortex (asterisk) in a cresyl violet-stained section. Scale bar = 238 μm. **(B)** Acute 48 h mortality of non-transgenic (non-tg) and tg neonatal mouse lines expressing human mutant genes causing human neurodegeneration such as Parkinson's disease (A53T-αSyn), amyotrophic lateral sclerosis (G93A-SOD1), and Alzheimer's disease (APP-PS1). Values shown are mean ± standard deviation (*n* = 40 mice/group). Asterisks denote significant difference (*p* < 0.001) compared to non-tg. **(C)** Motor performance (on running wheel) of 30-day-old mice subjected to sham procedure or CCI at postnatal day 7. Tg A53T-αSyn and G93A-SOD1 mice with CCI had significant deficits in running activity. Values are means ± standard deviation (*n* = 10/group). Asterisks denote (*p* < 0.01) compared to sham.

CCI (Figure 1A) was produced in postnatal day 7 (p7) mice using a protocol adapted from our earlier model of neonatal mouse open skull cortical trauma (33) and excitotoxic injury (34), except that the injury was induced by non-penetrating, closed-head, weight-drop. Mice were deeply anesthetized using isoflurane as described (33, 34). The contusion device consists of a pivotable hollow plastic cylinder 20 cm in length and a 5 mm diameter opening allowing for loading of 1 or 5 g weights. The angle of the skull impact was ~80°. Sham Tg and sham non-Tg mice were anesthetized and the weight-drop guide barrel and weight were placed gently on the skull without drop impact. Only male mice, identified by their external genitalia, were used. Each group initially had 40 mice.

### Clinical Outcome and Neurological Measurements

Acute spontaneous deaths occurring over the initial 48 h after injury were recorded. Morbidity was assessed by testing survivors on a voluntary running wheel at 30 days of age as described (27, 35, 36). Predetermined mouse survivals were 3 h, 24 h, 30 days, 3 months and endstage disease for lifespan determination. Animal attrition from the study was also noted over longer-term follow-up. For the 24 h time point, moribund mouse pups were excluded; pups with a milk spot were used. Onset of clinical disease of A53T-hαSyn mice was designated as the day the mouse shows slow ataxic and awkward jerking/spastic movements while walking (27). Endstage disease for A53T-hαSyn mice was defined as severe rigidity and bradykinesia that impairs movement to feed (27). Onset of clinical disease of G93A-hSOD1 mice was designated as the day the mouse shows hind-leg tremor (31, 32). Endstage disease for G93A-hSOD1 mice was defined as paralysis that impairs movement to feed (32).

### Brain Harvesting and Processing for Biochemistry and Histology

Naïve, sham, and CCI mice were killed at 3 and 24 h after the procedure, at 1 day, 30 days, and 3 and 6 months of age, or were euthanized at endstage disease. For the A53T-hαSyn cohort, mice at 24 h after CCI and 30 days of age were used for histology (*n* = 10/group) and biochemistry (*n* = 6/group), and mice at 3 months of age were used for biochemistry (*n* = 6/group). For the G93A-hSOD1 cohort, mice at 3 and 24 h after CCI were used for biochemistry (*n* = 6/group). For the hAPP-PS1 cohort, mice at 6 months of age after CCI were used for histology (*n* = 6/group). Mice were killed by an overdose of sodium pentobarbital. For biochemistry, the brains were removed rapidly from the skull after decapitation, divided into contralateral and ipsilateral hemispheres, and snap frozen in isopentane made cold (−78°C) by dry ice. For histology the mice were perfused through the heart with cold (4°C) phosphate buffer-saline (PBS, 100 mM, pH 7.4) followed by ice-chilled 4% paraformaldehyde. After perfusion-fixation, the brain was removed after 2 h, postfixed overnight in 4% paraformaldehyde (4°C), and cryoprotected 24 h in 20% glycerol-PBS (4°C). The fixed brains for A53T-hαSyn cohort were frozen under dry ice and were sectioned serially from frontal pole to posterior cerebellum in the coronal plane at 40 μm on a sliding microtome. The fixed brains for hAPP-PS1 cohort were frozen under dry ice and were sectioned serially from the lateral neocortical convexity to the midline in the sagittal plane at 40 μm on a sliding microtome. Every section was saved individually in 96-well plates containing antifreeze buffer. The sections were stored at −20°C. For histological analyses of A53T-hαSyn, brain sections were selected systematically and stained using cresyl violet (CV) for Nissl substance staining, cell morphology, and counting (27, 31), FD-silver (FD Neurotechnologies Inc., Baltimore, MD) for neurodegeneration (37), cytochrome c oxidase (COX) enzyme histochemistry for mitochondrial complex IV activity (38), and immunohistochemistry for mitochondrial morphology (31, 32). For histological analyses of hAPP-PS1 mice, brain sections were selected systematically and stained using immunoperoxidase immunohistochemistry for APP/amyloid β protein (Aβ).

### Electron Microscopy

Electron microscopy (EM) was used for ultrastructural assessments to confirm mitochondrial pathology in PD mice with neonatal CCI. From some brains from A53T-hαSyn mice (*n* = 3 sham, *n* = 3 CCI), neocortical samples were taken after perfusion-fixation and post-fixed in 2% glutaraldehyde. The samples were used to identify unequivocally mitochondrial swelling by EM as described (31, 32).

### Regional Forebrain Measurements and Cell Counting

Neocortical gray mantle and subcortical white matter thicknesses in A53T-hαSyn mice were measured by ocular filar micrometry in Nissl-stained brain sections at a level of bregma −0.58. Somatosensory (S1) cortex and corpus callosum were analyzed in five different sections in each mouse. Dorsal septal diameters were measured in the same anterior-posterior level in the same mice. Profile counting of Nissl-stained sections was done to estimate the numbers of neurons in the substantia nigra pars compacta (SNc) and pedunculopontine tegmental nucleus (PPN). Neurons in these regions were counted in anatomical level-matched sections (2–3 sections/region) at 1,000x magnification. Corresponding to a standard mouse brain stereotaxic atlas, SNc neurons were counted at bregma −292, −3.16, and −3.52; PPN neurons were counted at bregma −4.48 and −4.60. Strict morphological criteria were applied when classifying normal appearing neurons, including a round, open, euchromatic nucleus (not condensed and darkly stained), globular Nissl staining of the cytoplasm, clear vacuole-free cytoplasm, and a cell body diameter of ~10–20 μm. With these criteria, degenerating neurons with necrotic, apoptotic, and necrotic-apoptotic hybrids as well as astrocytes, oligodendrocytes, and microglia were excluded from the counts.

### Immunohistochemistry

We used superoxide dismutase-2 (SOD2) as an *in situ* mitochondrial marker (31, 32). SOD2 immunoreactive cytoplasmic particles have been used for assessing mitochondrial morphology such as mitochondrial diameters (31, 32, 39). Immunoperoxidase histochemistry with diaminobenzidine (DAB) as chromogen was used as described (31, 32, 39) to detect SOD2 protein with a highly specific rabbit polyclonal antibody (SOD-111, Stressgen). Mitochondrial diameters were measured in A53T-hαSyn mice by ocular filar micrometry in somatosensoty cortex and hippocampus CA1 in anatomical level-matched sections (2–3 sections/region) at 1,000x magnification. Immunoperoxidase histochemistry with DAB was used to detect Aβ-positive parenchymal deposits in hAPP-PS1 mouse neocortex with monoclonal antibody 6E10 (Covance) and CV-counterstaining. In mid-sagittal sections, all Aβ plaques were counted from frontal pole to posterior cingulate cortex.

### COX Activity

We used COX (complex IV) enzyme histochemistry as an *in situ* mitochondrial oxidative metabolism assay (40). This non-antibody based histological biochemical method is a functional assay that detects complex IV enxyme activity in tissue sections (40). The enzymatic reaction method has been described (31, 38, 41, 42). Brain regional enzyme activity was quantified by densitometry (31, 42, 43).

### Protein Oxidation Assay

Carbonylated proteins were detected with the OxyBlot Protein Oxidation Detection Kit (Millipore, Burlington, MA, USA) as described (44). Negative controls were prepared on paired brain homogenates by adding hydrazine derivatization solution or derivatization control solution (non-derivatized). Protein loading was measured by Ponceau S staining of the membrane used to detect carbonylated proteins. To quantify protein immunoreactivity, films were scanned and densitometry was performed as described (45). We used ImageJ to analyze the immunoreactive band intensities normalized to (divided by) the total protein in Ponceau S-stained gels. Protein levels were expressed as relative optical density measurements.

### Western Blotting

Western blotting was done to detect hαSyn and nitrated hαSyn (27). Crude tissue extracts were prepared from ipsilateral hemisphere forebrain. Protein fractions were subjected to SDS-PAGE and immunoblotting using enhanced chemilumesence detection as described (27, 32, 45). The reliability of sample loading and electroblotting in each experiment was evaluated by staining nitrocellulose membranes with Ponceau S before immunoblotting and by reprobing the blot for synaptophysin using a highly specific rabbit polyclonal antibody (Dako). If transfer was not uniform based on the Ponceau S, blots were discarded and gels were run again. Mouse monoclonal antibody clone Syn211 (46) was used to detect hαSyn. Mouse monoclonal antibody clone Syn12 (46) was used to detect nitrated synuclein. The antibodies were used at concentrations for visualizing protein immunoreactivity within the linear range.

### Statistical Analyses

All outcomes were assessed in a manner blind to treatment. After the procedure, all animals were coded so that subsequent experimenters were aware only of animal number. For histological and western blot measurements, group means, and variances were evaluated statistically by one-way ANOVA followed by a Newman-Keuls *post-hoc* test.

### Photography and Figure Construction

Marker comparisons between sham and CCI mice were made from sections that were imaged under identical conditions and analyzed using identical parameters. Original images used for figure construction were generated using digital photography. Digital images were captured as TiF files using a SPOT digital camera and SPOT Advanced software (Diagnostic Instruments) or a Nikon digital camera (DXM1200) and ACT-1 software. Images were altered slightly for brightness and contrast using ArcSoft PhotoStudio 2,000 or Adobe Photoshop software without changing the content and actual result. Figure composition was done using CorelDraw software with final figures being converted to TiF files. Files of composite figures were adjusted for brightness and contrast in Adobe Photoshop.

## Results

### Adult-Onset Neurodegenerative Disease Gene Mutations Can Worsen Acute and Delayed Clinical Outcome in Mice After Neonatal Traumatic Cortical Injury

This TBI model produces a frontal-parietal-occipital cortical lesion depending on the intended placement of the weight-guide cylinder (Figure 1A, arrow). The mice in this study had frontal-parietal injury. In non-tg mice, the acute insult is maximally mild as evidenced by the ~90% survival (Figure 1B). CCI on neonatal mutant A53T-hαSyn and mutant G93A-hSOD1 tg mice causes significantly greater mortality (*p* < 0.01) than in injured non-tg mice (Figure 1B, *n* = 40 males/genotype). Similar CCI on a mouse model of AD (APP/PS1ΔE9 line) did not cause significant effects on acute mortality (Figure 1B) nor was there any mouse attrition over longer periods. Morbidity after neonatal CCI was manifested in juveniles as evidenced by the significant deficit in motor activity in mutant A53T-hαSyn tg mice and mutant G93A-hSOD1 tg mice compared to respective age-matched sham mutant mice (Figure 1C).

### Parkinson's Disease Causing Mutant α-Synuclein Exacerbates Brain Damage After Neonatal TBI

**Figure 2:**
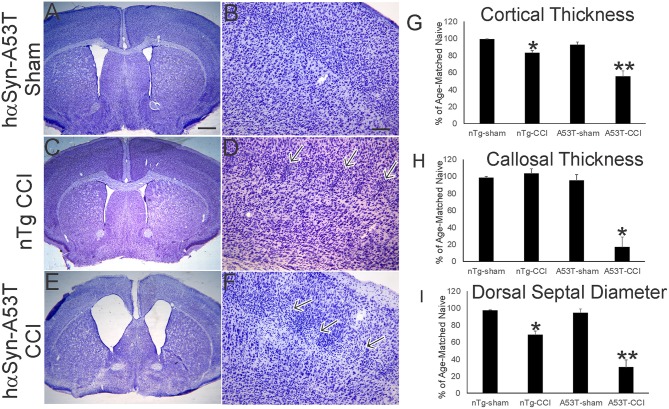
Mutant A53T-hαSyn exacerbates forebrain injury in a neonatal mouse model of closed skull cortical contusion injury (CCI). Mice received CCI on postnatal day 7 and then killed at postnatal day 30 to show forebrain histology by cresyl violet staining. Shown are anatomically-matched levels of forebrain from Tg A53T-hαSyn mice with sham procedure **(A,B)**, non-Tg (nTg) mice with CCI **(C,D)**, and Tg A53T-hαSyn mice with CCI **(D,E)**. **(A,B)** Thirty-day-old sham Tg A53T-hαSyn mice have histologically normal cerebral cortex, septum, and striatum. No abnormalities in cortical lamination and cytology are apparent **(B)**. **(C,D)** Thirty-day-old nTg mice with CCI have slightly smaller forebrains with a modest reduction in cerebral cortical thickness **(G)**, and septal atrophy **(I)**, but no thinning of corpus callosum **(H)**. In the middle layers of cerebral cortex, some foci of small cell accumulation were present (**D**, arrows). **(E,F)** Thirty-day-old Tg A53T-hαSyn mice with CCI have grossly atrophic forebrains **(E)** as evident by the dilation of the lateral ventricles, marked cerebral cortical and corpus callosum thinning **(G,H)**, and prominent atrophy of septum **(I)**. In the middle and superficial layers of cortex, discontinuous patchy pale zones of apparent cell loss are present (**F**, arrows). **(G)** Cortical thickness (vertical depth, surface of cortex to start of corpus callosum) in sham and CCI mice at postnatal day 30. Measurements were made in somatosensory cortex. Values are mean ± SD (*n* = 10 mice/group). ^*^p < 0.01 compared to non-Tg sham; ^**^p < 0.005 compared to A53T-sham. **(H)** Corpus callosum thickness (vertical depth, inferior cortical layer 6 end to striatum) in sham and CCI mice at postnatal day 30. Measurements were made deep to somatosensory cortex. Values are mean ± SD (*n* = 10 mice/group). ^*^p < 0.001 compared to A53T-sham. **(I)** Diameter of the dorsal septum in sham and CCI mice at postnatal day 30. Measurements were made precisely inferior to the decussation of the corpus callosum. Values are mean ± SD (*n* = 10 mice/group). ^*^p < 0.01 compared to non-Tg sham; ^**^p < 0.005 compared to A53T-sham. Scale bars: A (same for **C,E**) = 476 μm; **B** (same for **D,F**) = 83 μm.

Nissl staining (Figure 2) was used to characterize forebrain neuropathology in this model. We focused on the mutant hαSyn mouse because of the significant acute neonatal mortality and because head trauma is a known risk factor for developing PD in human (9, 10). In neonatal rodent brain injury models, sham surgical procedures are essential because of the potential of anesthesia toxicity (47), and even making a skull bone window with the utmost care can cause damage (33). Here, sham surgeries on p7 mice involved anesthesia and placement of the weight and guide barrel on the head without drop impact. The brain damage seen at p30 after p7 CCI is reproducible. In non-tg mice, the insult causes maximally mild injury as reveal by the ~10–20% reduction in sensorimotor cortical thickness (Figure 1A, inset, Figures 2C,D,G), but apparent small cell inflammatory changes are persistent (Figure 2D, arrows). The cerebral cortex of sham A53T-hαSyn mice was unremarkable at this age (Figures 2A,B,G) consistent with other work (27). Cortical atrophy in p30 A53T-hαSyn mice with p7 CCI was severe compared to non-tg mice with CCI and sham A53T-hαSyn mice (Figures 2E,G), and foci of attenuated cell density were evident (Figure 2F). Severe forebrain pathology was also evident in A53T-hαSyn mice with CCI by significant reductions in corpus callosum thickness (Figure 2H) and septal atrophy (Figure 2I).

**Figure 3:**
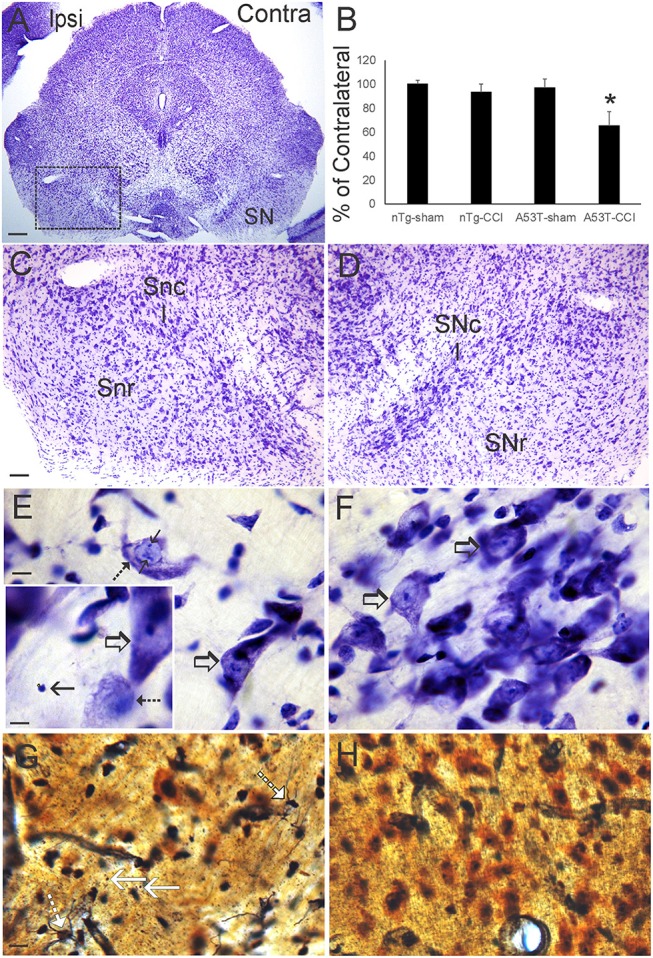
Neonatal cortical contusion injury (CCI) causes early degeneration of the substantia nigra. **(A)** Cresyl violet-stained section of midbrain from a 30-day-old mutant A53T-hα Syn mouse with CCI on postnatal day 7. Ipsilateral (ipsi) is the side with the CCI, and contralateral (contra) is the side without the CCI. Box delineates part of the substantia nigra (SN) shown at higher magnification in **(C,D)**. **(B)** Counts of large Nissl-stained neurons in the substantia pars compacta (SNc) at 1 month of age after CCI at postnatal day 7. Values are mean ± SD (*n* = 10 mice/group). ^*^p < 0.01 compared to all other groups. **(C,D)** Nissl staining of the SN in ipsi **(C)** and contra **(D)** sides of the ventral midbrain after CCI. On the control Contra side **(D)** the SNc is clearly divisible from the substantia nigra reticulata (SNr). In the Ipsi SNc **(C)**, cell loss is apparent particularly in the central SNc. (**E,F)** High magnification of the central SNc on Ipsi **(E)** and Contra **(F)** sides. Cell loss is apparent ipsilaterally **(E)** compared to the contralateral SNc **(F)** that is populated by large healthy-appearing multipolar neurons (**F**, open arrows). Ongoing neuron degeneration is present in the ipsilateral SNc as demonstrated by a large neuron with chromatolytic features (**F**, hatched arrow) and nuclear inclusions (**F**, solid arrow). A nearby neuron appears normal (**E**, open arrow). Inset in **(F)** shows an endstage apoptotic profile (solid arrow), a degenerating neuron with chromatolytic features and condensed nucleus (hatched arrow), and a normal neuron (open arrow). **(G,H)** Silver staining in the SNc shows degenerating axons with neuritic abnormalities/degenerating cell bodies (**G**, hatched white arrows) and degenerating axon terminals (**G**, solid white arrows). Degenerating profiles were not seen in the contralateral SNc **(H)** in the same sections. Scale bars: **(A)**, 250 μm; **(C)** (same for **D**) 100 μm; **(E)** (same for **F**) 10 μm; **(E)** (inset) 8 μm; **(G)** (same for **H**) 12.5 μm.

**Figure 4:**
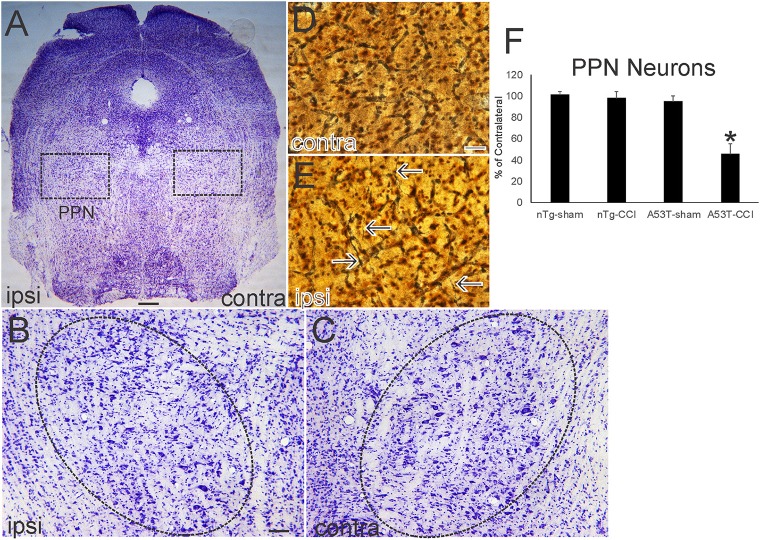
Neonatal cortical contusion injury (CCI) causes early degeneration in brainstem. **(A)** Cresyl violet-stained section of near-pontine brainstem from a 30-day-old mutant A53T-hαSyn mouse with CCI on postnatal day 7. Ipsilateral (ipsi) is the side with the CCI, and contralateral (contra) is the side without the CCI. Boxes delineate bilaterally the pedunculopontine tegmental nucleus (PPN) shown at higher magnification in **(B,C)**. **(B,C)** The ipsi **(B)** and contra **(C)** PPN are delineated by the hatched oval. The ipsi PPN shows apparent loss of large neurons. (**D,E)** Silver staining in the ipsi PPN **(E)** shows degenerating axons with neuritic abnormalities (**E**, arrows). Degenerating profiles were not seen in the contra PPN **(D)**. **(F)** Counts of large Nissl-stained neurons in the PPN at 1 month of age after CCI at postnatal day 7. Values are mean ± SD (*n* = 10 mice/group). ^*^p < 0.01 compared to all other groups. Scale bars: **(A)**, 322 μm; **(B)** (same for **C**), 100 μm; **(D)** (same for **E**) 12.5 μm.

Nissl and silver staining revealed ventral midbrain and brainstem neuropathology in this neonatal CCI model at 1 month of age. Neuronal loss (~30%, *p* < 0.01) was found in the ipsilateral substantia nigra pars compacta (SNc) in A53T-hαSyn mice with CCI but not in age-matched sham A53T-hαSyn mice or in non-Tg mice with CCI (Figures 3A–D). Some remaining ipsilateral SNc neurons showed chromatolytic reaction, consistent with axonopathy (33, 41, 48), or cytoplasmic palor and cell nucleus abnormalities (Figure 3E) compared to the contralateral SNc neurons seen as normal large multipolar, Nissl-rich profiles (Figure 3F). Apoptotic profiles were also observed in the ipsilateral SNc in A53T-hαSyn mice with CCI (Figure 3E inset) but not contralaterally. Silver staining delineated degenerating neuronal cell bodies, axons, and terminals in the ipsilateral SNc of A53T-hαSyn mice with CCI (Figure 3G) but not in the contralateral SNc (Figure 3H) or in 1-month-old sham A53T-hαSyn mice or non-Tg mice with CCI. In the brainstem tegmentum, the pedunculopontine tegmental nucleus (PPN) of A53T-hαSyn mice with CCI had ~50% loss of neurons ipsilaterally compared to the contralateral PPN (Figures 4A–C,F). No loss of PPN neurons was detected in age-matched sham A53T-hαSyn mice or in non-Tg mice with CCI (Figure 4F). Silver staining identified axonal and neuritic pathology in the ipsilateral PPN (Figure 4E) but not in the contralateral PPN (Figure 4D) or in the PPN of age-matched sham A53T-hαSyn mice or non-Tg mice with CCI. In contrast to the SNc and PPN, the red nucleus, raphae nucleus, and bulbar cranial nerve motor neuron groups were all unremarkable (data not shown).

### Mutant α-Synuclein Exacerbates Protein Oxidative Damage in Forebrain After Neonatal TBI

**Figure 5:**
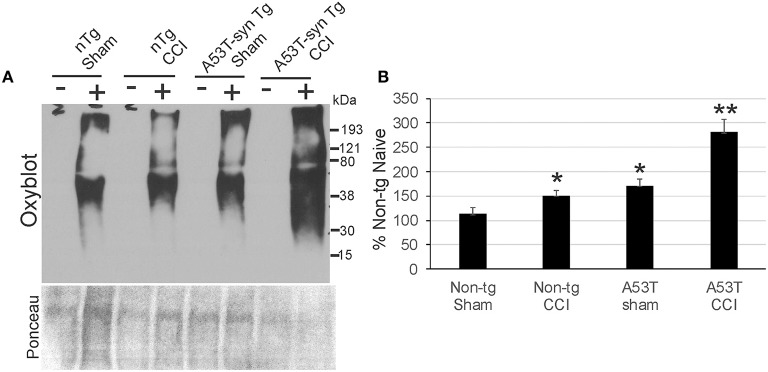
Protein oxidation is exacerbated acutely in mutant A53T-αSyn after neonatal CCI. **(A)** Oxyblot showing protein carbonyls in forebrain of sham and cortical contusion injury (CCI) mice 24 h after injury at postnatal day 7. Plus (+) and minus (–) lane designations are same samples with and without (negative control) chemical derivatization. Ponceau S-stained membrane shows protein loading for each lane. **(B)** Densitometry measurements of total-lane protein carbonyl immunoreactivities in sham and CCI mice. Values are mean ± SD (*n* = 6 mice/group). ^*^p < 0.01 compared to non-Tg sham; ^**^p < 0.001 compared to A53T-sham.

Protein oxidative damage in mouse cerebrum at 24 h after CCI was assessed by the levels of carbonyl-modified proteins after derivatization and western blotting. Carbonylated proteins were detected at 15–250 kD throughout the length of the gels (Figure 5A). Negative controls without protein derivativation but with exposure to dinitrophenylhydrazone antibody were blank (Figure 5A). Non-tg CCI mice and A53T-hαSyn mice sham mice differed modestly but significantly (*p* < 0.05) from non-tg shams (Figure 5B). In contrast, protein carbonyl levels were dramatically elevated in A53T-hαSyn mice with CCI compared to non-tg sham (*p* < 0.001) as well as non-tg CCI and sham A53T-hαSyn mice (*p* < 0.01). Interestingly, the basal pattern of oxidized proteins appeared preserved rather than sets of apparently different proteins emerging with carbonylation after injury.

### Mutant α-Synuclein Mice Have Elevated Mitochondrial Oxidative Activity Acutely After Neonatal TBI

**Figure 6:**
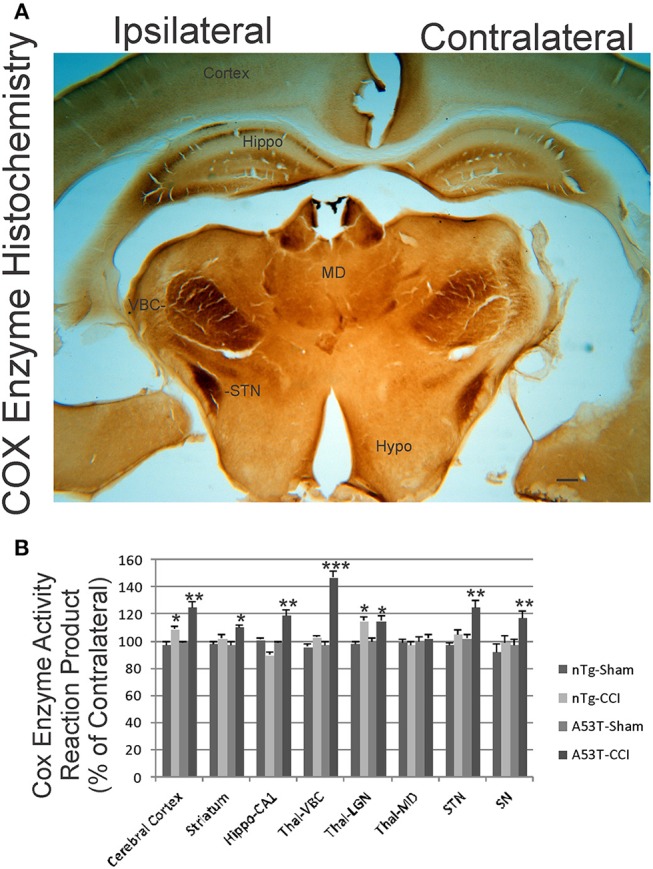
Mitochondrial oxidative metabolism is amplified acutely in mutant A53T-αSyn after neonatal cortical contusion injury (CCI). **(A)** Cytochrome c oxidase (COX) enzyme histochemistry showing the *in situ* enzyme activity of COX in neonatal mouse brain at 24 h after injury at postnatal day 7. The ventrobasal complex of thalamus (VBC), subthalamic nucleus (STN), and hippocampus (Hippo) show intense mitochondrial activation ipsilaterally after CCI. The mediodorsal thalamic nucleus (MD) and hypothalamus (Hypo) are unchanged and similar on both hemispheres. Scale bar = 255 μm. **(B)** Densitometry measurements of COX enzyme activity reaction product in sham and CCI mice. Values are mean ± SD (*n* = 10 mice/group). ^*^p < 0.05 compared to non-Tg sham; ^**^p < 0.01, or ^***^p < 0.001 compared to A53T-sham.

We used COX enzyme histochemistry to detect mitochondrial metabolic activity *in situ*. The specificity of this histochemical reaction for COX has been established (40, 42). As shown before (40, 43), the brain regional distribution of COX activity varies widely at baseline (Figure 6, contralateral side). The thalamic ventrobasal nuclear complex and subthalamic nucleus show high activity, while other areas of diencephalon have lower activity (Figure 6A). The distinct layers of hippocampus show differential activity, with the CA1 stratum lacunosum-moleculare showing the highest activity and the stratum radiatum and stratum pyramidale with lower activity (Figure 6A). At 1 day after CCI, the ipsilateral hemisphere in non-tg mice had modest, but significant, elevations in mitochondrial activity in cerebral cortex and the thalamic lateral geniculate nucleus. In A53T-hαSyn mice with CCI, the enhancement of mitochondrial oxidative metabolism was exacerbated in magnitude and regional distribution with the thalamic ventrobasal complex showing the greatest activation (Figures 6A,B). The pyramidal cell layer of hippocampus also had a marked increase in COX activity in A53T-hαSyn mice with CCI at 1 day recovery (Figures 6A,B).

### Mutant α-Synuclein Mice Accumulate Swollen Mitochondria Acutely After Neonatal TBI

**Figure 7:**
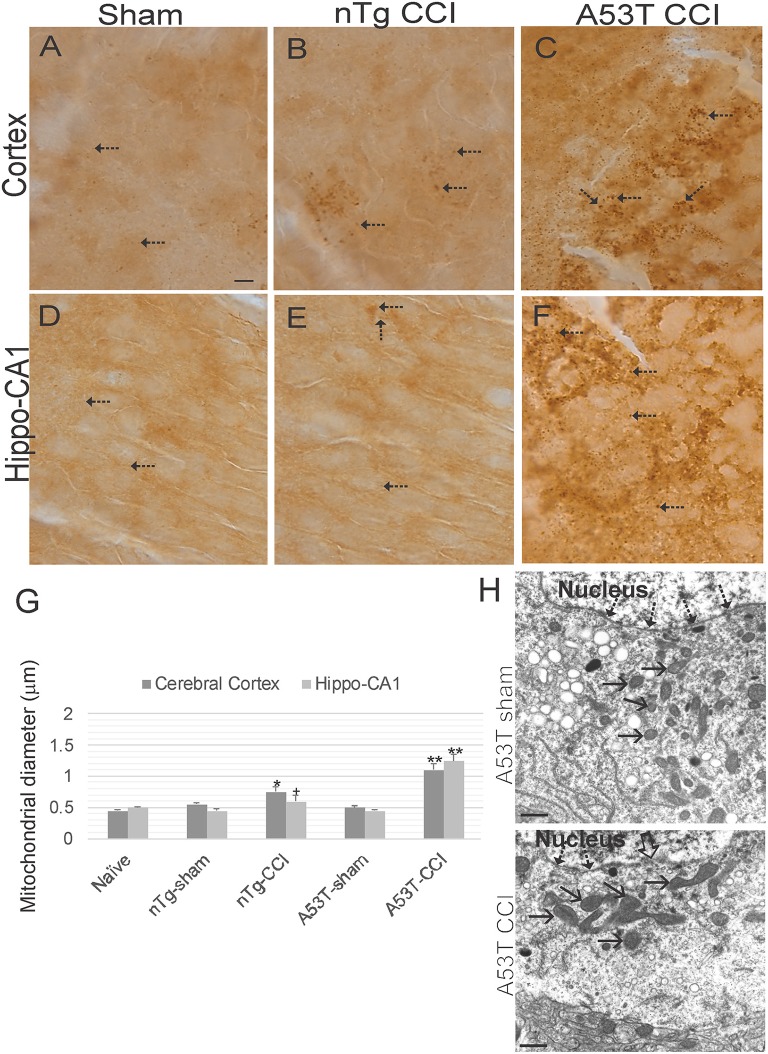
Mitochondrial morphology abnormalities in forebrain are exacerbated in mutant A53T-αSyn after neonatal cortical contusion injury (CCI). SOD2 immunostaining showing mitochondria in neonatal mouse cerebral cortex **(A–C)** and hippocampus **(D–F)** at 24 h after injury at postnatal day 7. **(A–C)** In cerebral cortex mitochondria in shams were fine and barely visible (by light microscopy) speck-like particles dispersed throughout the neuropil (**A**, arrows). In non-Tg mice after CCI clusters of larger swollen mitochondria were observed, (**B**, arrows), and in mutant A53T-hαSyn mice with CCI large swollen mitochondria were found commonly throughout the cortical neuropil (**C**, arrows). **(D–F)** In hippocampus swollen mitochondrial were most conspicuous in mutant A53T-αSyn mice with CCI (**F**, arrows) though some isolated cells appeared with clusters of swollen mitochondria in non-Tg mice with CCI. Scale bar A (same for **B–F**) = 10 μm. **(G)** Mitochondrial diameter measured (1,000x) in cross-sectional profiles in hippocampus and cerebral cortex at 24 h after injury at postnatal day 7. Values are mean ± SD (*n* = 10 mice/group). +*p* < 0.05, ^*^p < 0.01, or ^**^p < 0.005 compared to sham. **(H)** EM of ipsilateral cortical neurons at 24 h after sham or CCI procedure on mutant A53T-hαSyn mice. Hatched arrows identify the nuclear membrane. Open arrow (lower panel) identifies breach in the nuclear membrane of CCI mouse cortical neuron. Solid arrows identify cross-sectional profiles of mitochondria in the perikaryon. Mitochondria are swollen in the neuron of CCI mice. Scale bars = 1 μm.

Immunohistochemistry for SOD2 (Figures 7A–F) was used as an additional assay to study mitochondria and was used to measure mitochondrial diameters as an index of their swelling directly in neurons (31, 32, 39). In naïve wildtype mice, mitochrondrial diameter in various neurons is about 0.5 μm (Figures 7A,D,G). Both sham groups were no different from naïve mice (Figure 7G). In contrast, mitochondrial diameters in cortical and hippocampal neurons were modestly increased in non-Tg mice with CCI at 1 day recovery (Figures 7B,E,G). Mitochondrial swelling at 24 h after CCI was markedly exacerbated in A53T-hαSyn mice (Figures 7C,F,G). Mitochondrial swelling in cortical neurons was confimed by EM (Figure 7H).

### Acute Brain Injury in Early Life Accelerates Later Development of PD in hαSyn tg Mice

**Figure 8:**
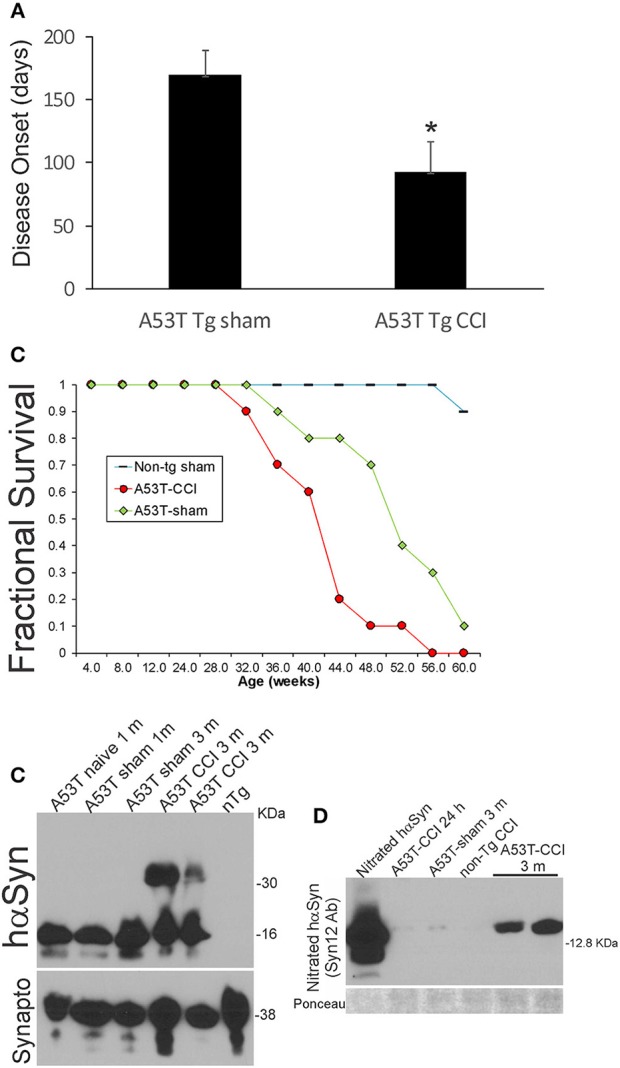
Early-life brain trauma accelerates the development of later-life PD and proteinopathy in mutant A53T-αSyn Tg mice. **(A)** PD-like disease onset occurs significantly (^*^p < 0.01) earlier in mutant A53T-αSyn Tg mice with postnatal day 7 CCI compared to sham-procedure mutant A53T-αSyn Tg mice. Values are mean ± SD (*n* = 10 mice/group). **(B)** Kaplan-Meier plot showing that PD-like disease onset, neurological decline, and death occur earlier in mutant A53T-αSyn Tg mice with postnatal day 7 CCI compared to sham-procedure mutant A53T-αSyn Tg mice. **(C)** Western blotting shows advanced hαSyn oligomerization in forebrain at 3 months of age in mutant A53T-αSyn Tg mice with postnatal day 7 CCI compared to naïve and sham-procedure mutant A53T-αSyn Tg mice. Synaptophysin (Synapto) immunoblot shows protein loading. **(D)** Western blotting shows advanced hαSyn nitration in forebrain at 3 months of age in mutant A53T-αSyn Tg mice with postnatal day 7 CCI compared to age-matched sham-procedure mutant A53T-αSyn Tg mice and mutant A53T-αSyn Tg mice with CCI at 24 h. Ponceau S-stained membrane shows protein loading.

Neonatal hαSyn-A53T tg mice with p7 CCI developed motor deficits at a juvenile age compared to age-matched non-injured sham hαSyn tg-A53T mice (Figure 1C). Disease onset, defined by the presence of jerky/spastic movements [(27), see videos], was accelerated in hαSyn-A53T tg mice with CCI (Figure 8A). Furthermore, hαSyn-A53T tg mice with neonatal CCI had a shortened lifespan compared to age-matched non-injured sham hαSyn tg-A53T mice (Figure 8B). The early disease onset at about 3 months of age in hαSyn-A53T tg mice with neonatal CCI was associated with prominent accumulation of nitrated hαSyn and hαSyn oligomers in brain (Figures 8C,D).

### Mutant hSOD1 Causes Severe Protein Oxidative Damage in Forebrain After Neonatal TBI

**Figure 9:**
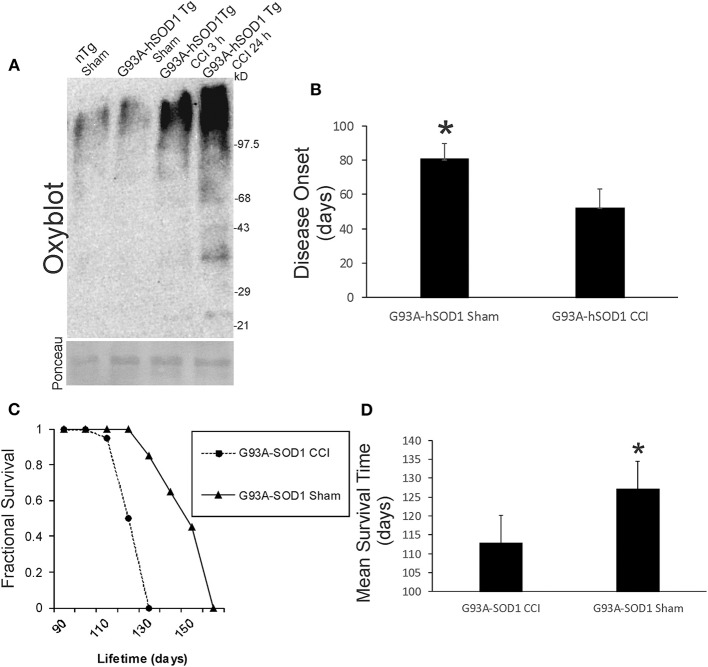
Early-life brain trauma in ALS mice causes severe oxidative damage and accelerates the development of later-life ALS in G93A-hSOD1 Tg mice. **(A)** Oxyblot showing protein carbonyls in forebrain of sham and cortical contusion injury (CCI) mice 3 and 24 h after injury at postnatal day 7. Molecular weight standards (in kD) shown at right. Ponceau S-stained membrane shows protein loading for each lane. **(B)** Neonatal CCI at p7 in G93A-hSOD1 Tg mice causes an earlier disease onset compared to tg mice without CCI. Values are mean ± SD (*n* = 10 mice/group). Asterisk significantly (*p* < 0.05) later compared to CCI. **(C)** Kaplan-Meier plot showing that ALS-like disease onset, neurological decline, and death occur earlier in G93A-hSOD1 Tg mice with postnatal day 7 CCI compared to sham-procedure G93A-hSOD1 Tg mice. (**D)** Graph showing the mean survial of G93A-hSOD1 Tg mice with and without CCI. Values are mean ± SD (*n* = 10 mice/group). Asterisk significantly (*p* < 0.05) later compared to CCI.

We examined acute oxidative stress after neonatal CCI in our ALS mouse model. Protein oxidative damage in mouse cerebrum at 3 and 24 h after CCI was assessed by the levels of carbonyl-modified proteins after derivatization and western blotting. At baseline, carbonylated proteins were detected at the high molecular weight range 150–250 kD (Figure 9A), and non-tg sham and G93A-hSOD1 sham mice had similar patterns. Baseline protein carbonyl patterns were very different in hαSyn-A53T tg mice and their non-tg sham controls compared to G93A-hSOD1 tg mice and their non-tg sham controls probably because these mouse lines have completely different genetic background strains. After CCI, protein carbonyl levels were dramatically elevated in G93A-hSOD1 compared to non-tg sham at 3 h (Figure 9A). At 24 h after CCI the accumulation of protein carbonlys was greater compared to 3 h with very distinct bands of damaged protein bands resolving in the lower- to mid-molecular weight ranges (Figure 9A).

### Acute Brain Injury in Early Life Accelerates Later Development of ALS in G93A-hSOD1 tg Mice

Neonatal G93A-hSOD1 tg mice with p7 CCI developed motor deficits at a juvenile age compared to age-matched non-injured sham hαSyn tg-A53T mice (Figure 1C). Disease onset, defined by the presence of leg tremor was significantly earlier in G93A-hSOD1 tg mice with CCI (Figure 9B). G93A-hSOD1 tg mice with neonatal CCI also had a significantly abbreviated lifespan compared to age-matched non-injured sham G93A-hSOD1 tg mice (Figures 9C,D). G93A-hSOD1 tg mice with neonatal CCI died about 15 days earlier than their tg sham controls (Figure 9D).

### Acute Brain Injury in Early Life Did Not Affect the Deposition of Amyloid in APP-PS1 tg Mice

**Figure 10:**
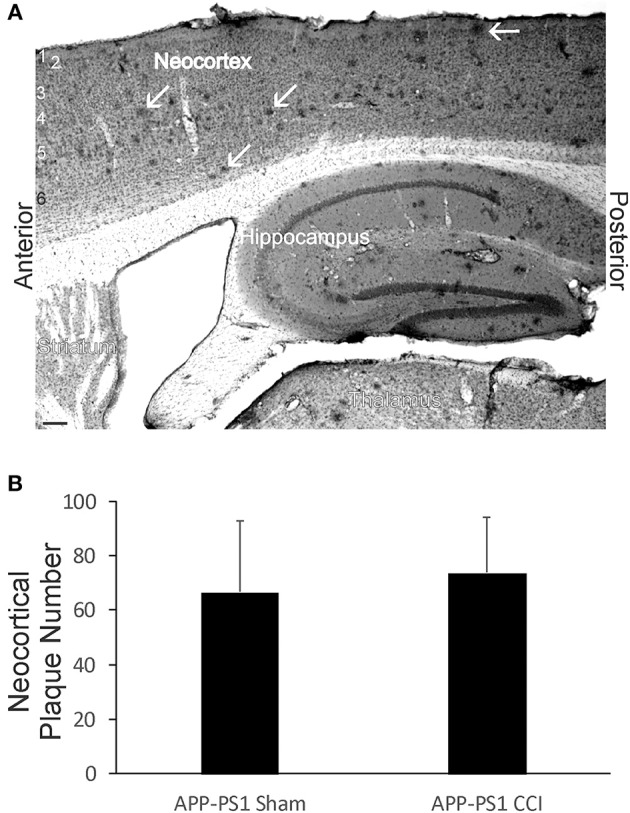
Early-life brain trauma in APP-PS1 mice does not alter the cortical accumulation of Aβ. **(A)** Sagittal section stained immunohistochemically for Aβ. Cortical layers identified by numbers (left). White arrows identify parenchymal deposits of Aβ. Bar = 103 μm. **(B)** Graph showing the mean number of Aβ deposits in frontal-patietal-occipital cortex in APP-PS1 Tg mice with and without CCI. Values are mean ± SD (*n* = 6 mice/group).

Although early life brain injury did not after acute mortality (Figure 1B) or apparent morbidity in APP-PS1 mice, we assayed for later-life brain pathology by counting the number of neocortical Aβ deposits at 6 months of age. Neonatal APP-PS1 tg mice with p7 CCI did not accumulate more Aβ deposits compared to age-matched non-injured sham APP-PS1 mice (Figure 10).

## Discussion

This study shows that acute outcome after acquired brain injury in newborns is influenced significantly by mutant genes that cause adult-onset neurodegenerative disease and that the development of later-life age-related neurodegenerative disease is influenced significantly by early-life brain injury. Specifically, genes causing PD and ALS worsen mortality and morbidity in mice with neonatal CCI. Reciprocally, mice genetically predisposed to develop PD or ALS later in life have accelerated disease when they have early-life TBI. Worse acute outcome in PD- and ALS–destined mice with neonatal TBI is associated with exacerbated oxidative damage. More severe mitochondriopathy is also seen in PD mice with neonatal TBI. Accelerated later-life disease is associated with exacerbated PD-related proteinopathy. Thus, in experimental settings, mutant genes that manifest their clinical phenotype in adulthood can have major impact on acquired CNS injury in infancy and, perhaps, childhood. From this study, it seems evident that neonatal brain injury and adult-onset neurodegeneration are more interrelated and intersecting than realized previously.

Precedent exists for genetic factors influencing acute and long-term neurologic outcome after acquired brain injury in human adults, children, and newborns. For example in adults, *neuroglobin* gene haplotype has a strong influence on outcome after TBI (49), *bcl-2* gene single nucleotide polymorphisms also influence outcome after TBI (50), and a*poE* genotype predicts outcome following subarachnoid hemorrhage (51). In children, single nucleotide polymorphisms in genes related to dopamine neurotransmission (D2 dopamine receptor, dopamine transporter, catechol-o-methytransferase, and ankyrin repeat and kinase domain containing (1) have roles in short- and long-term neurobehavioral recovery after TBI (20). In term newborns, c*atalase* gene (21) and *interleukin-6* gene (22–24) polymorphisms are associated with higher susceptibility to cerebral palsy after hypoxia-ischemia. In preterm infants, glial glutamate transporter gene *EAAT2* polymorphisms associate with greater risk of cerebral palsy (25). The novel information revealed in our study is the demonstration that pathogenic genes causing adult-onset neurodegenerative can have dramatic acute and long-term impacts on neonatal mice with acquired brain injury.

Pathogenic mechanisms of mutant genes that cause neurodegeneration in adults thus appear to influence brain responses to acute injury long before the spontaneous development of age-related neurodegenerative disease. In hαSyn-A53T tg mice with neonatal CCI, we found exacerbated protein oxidation and mitochondrial damage compared to non-tg neonatal CCI mice at 1-day of recovery. Moreover, hαSyn oligomerization and nitration were accelerated after early-life brain injury. In previous studies of a mouse model of ALS, acute peripheral nerve avulsion induces rapid spinal motor neuron necrosis rather than the usual apoptosis of motor neurons seen in non-tg mice (52), and, in this study, we found severe protein oxidative damage in neonatal ALS mice with brain injury. Electrophysiological and ion channel activities of cultured embryonic motor neurons (53, 54) and early postnatal motor neurons in acute spinal cord slices (55) from a mutant hSOD1 mouse model of ALS are abnormal long before these mice show a clinical phenotype or overt neuronal cell death. Similarly, mitochondriopathy exists before clinical features and neurodegeneration emerge in these mice (31, 54, 56). Thus, individuals with “adult-onset” neurodegenerative disease may be primed or predisposed to have worse clinical and pathological outcomes after early-life CNS injury. It would be interesting to know if infants and children that have unexplained poor outcomes in intensive care units have genetic risk factors such a mutant genes that would eventually cause PD and ALS.

The influences of the human mutant genes on outcome after neonatal brain injury in mice were manifested acutely and over long-term. Mice harboring hαSyn-A53T and hSOD1-G93A mutations had significantly worse 48-h survival after CCI. The hαSyn-A53T transgene is driven by a Thy-1 neuron-specific promoter (27) and the hSOD1-G93A transgene is driven by the endogenous hSOD1 promoter (28), but, while the hαSyn has a much more restricted tissue expression of the mutant protein compared to the hSOD1 mice, there could be critical autonomic and peripheral responses after the insult unaccounted for in both mouse lines. The mice were not be instrumented and monitored for clinically relevant physiology, as in neonatal large animal models (57). In this regard, we do not know the physiological basis of why hαSyn-A53T and hSOD1-G93A mice had greater risk of death after acute brain injury, but it appears unrelated to their essential Tg nature because mice harboring human APP and PS1 mutant genes had survival rates similar to non-Tg mice. In the injured 24 h-surviving hαSyn-A53T mice, we observed exacerbated protein oxidation and mitochondrial injury. In G93A-hSOD1 mice with neonatal TBI severe protein oxidation was observed at 3 and 24 h after the insult. It is possible that this pathology contributed to their early deterioration and could be mechanisms related to the “priming” predisposition of age-related disease in these mice. At 1-month of age, clinical motor deficits and neuropathology in cerebral cortex, white matter, midbrain, and brainstem of hαSyn-A53T mice with neonatal TBI were significantly more severe than in non-Tg mice with CCI and sham hαSyn-A53T. Later in life hαSyn-A53T mice with neonatal TBI manifested much earlier onset of PD-like symptoms and eventiually developed fatal disease much earlier than sham hαSyn-A53T mice. The accelerated disease onset occurred in the presence of greater hαSyn proteinopathy. A similar acceleration of disease onset and death was seen in ALS mice with neonatal brain injury. Perhaps early-life brain injury, starting with the initial p7 mild traumatic damage to cerebral cortex, triggers seeding effects on synucleinopathy or hSOD1 misfolding that spreads in a connectivity-related prion-like mechanism that damages vulnerable regions and nuclei throughout the neuraxis. New hypotheses for PD and ALS pathogenesis describe prion-like (prionoid) spreading of abnormal proteins (38), thus accounting for the progression and staging of neuropathology throughout the nervous system in preclinical individuals through advanced disease (58).

Mutations in αSyn cause some forms of familial PD (5, 6). Aggregated and nitrated hαSyn forms are toxic entities in familial and sporadic forms of PD. αSyn is an abundant soluble monomeric protein that can associate with mitochondrial membranes (37, 59–61), and the pathogenesis of hαSyn-linked PD in mice involves the association hαSyn with mitochondria and activation of the mitochondrial permeability transition pore (27, 37). We found exacerbated mitochondrial damage in neonatal hαSyn-A53T mice with TBI compared to neonatal sham hαSyn-A53T mice. αSyn can polymerize into insoluble fibrils due to a conformational change from an α-helical coil to a β-pleated sheet (62). We found exacerbated aggregation of mutant hαSyn in neonatal hαSyn-A53T mice with TBI compared to neonatal sham hαSyn-A53T mice. hαSyn mutations cause increased levels of protofibrils, possibly being the more toxic form of the protein (63). hαSyn protofibrils might also be toxic by making membranes of cells more porous (64). Of particular relevance to our experimental model is that hαSyn fibrils are transported in neurons anterogradely and retrogradely, and they appear to propagate after transport and undergo neuron-to-neuron transmission independently of synapses and cell-to-cell-contacts (18). We found remote degeneration in the SNc and PPN in young hαSyn-A53T mice after CCI. Nitration of hαSyn, footprinting the presence of potent reactive nitrogen species like peroxynitrite, is a major signature of human PD and other proteinopathies and might be critical to the aggregation, toxicity, and spreading processes (46, 65). We found elevated hαSyn nitration in neonatal hαSyn-A53T mice with TBI compared to neonatal sham hαSyn-A53T mice. Aggregation and nitration of wildtype and mutated hαSyn is associated with enhanced cell death in cultured cells (46). We found exacerbated neuropathology in mutant hαSyn in neonatal hαSyn-A53T mice with TBI compared to neonatal non-Tg mice with TBI and sham hαSyn-A53T mice. Over-expression of wildtype or mutant hαSyn in cultured cells elevates the generation of intracellular reactive oxygen species and causes mitochondrial deficits (66, 67); moreover, expression of mutant hαSyn increases cytotoxicity to dopamine oxidation products (68). We found elevated protein oxidation in neonatal hαSyn-A53T mice with TBI compared to neonatal sham hαSyn-A53T mice. This precedent on hαSyn molecular pathology and our new findings suggest that acquired brain injury in early life can engage rapidly the mechanisms of age-related pathogenesis in neurodegenerative disease for acute responses, even in the immature brain, and then sustain or perpetuate these mechanisms to influence later-life disease outcomes.

In our study, we did not find an effect of human mutant APP and PS1 on acute outcome in injured neonatal mice. This is not surprising considering the limited age-related clinical phenotypes and neuropathology in this mouse model (4, 30). While these mice accumulate with aging large quantities of Aβ protein in brain, they do not develop fatal neurological disease or any major neurodegeneration, as defined by neuronal cell and synaptic loss, neurofibrillary tangle, and tauopathy (30, 69–71); thus, modeling ineffectively that seen in human AD (72–75). In this study, we did not find an effect of neonatal CCI on later-life Aβ deposition in neocortex. Despite the Aβ proteinopathy, overt mitochondrial pathology and protein oxidative damage are minimal in APP/PS1 Tg mice (30, 76) in comparision to that seen in hαSyn-A53T and G93A-hSOD1 mice (27, 32). Thus, it could be the robust bioenergetic and oxidative stress pathology in the hαSyn-A53T and G93A-hSOD1 mice that is recruited acutely and driving the deleterious responses of the immature brain to acquired injury. Neonatal brain injury in human tau Tg mice might yield more provocative acute and long-term results.

This study has limitations. We did not have available hαSyn-wildtype and hSOD1-wildtype mouse pups subjected to CCI for a comparison with the hαSyn-A53T and hSOD1-G93A mice with CCI. This comparison would have been useful to see if the hαSyn and hSOD1 overexpression *per se* was harmful or whether the detelerious effects were related to potential toxic gains in protein function due to the mutations. However, in an adult mouse model of cortical injury-induced target deprivation, wildtype hSOD1 overexpression afforded significant protection against retrograde neuronal apoptosis (45). We also did not compare male to female mouse responses. We have observed sex differences in neonatal mice after cerebral hypoxia-ischemia subacutely and over long-term (77) and in adult G93A-hSOD1 mice (32). We are also uncertain whether the apparent neuronal degeneration and loss in this mouse model mirrors that seen in human neurons (78).

## Ethics Statement

All animal studies were performed in accordance with institutional guidelines and laws of the United States of America. No research in this study involved human participants.

## Author Contributions

LM conceived and designed experiments, performed experiments, analyzed data, wrote paper. MW performed experiments and analyzed data. AH performed experiments and worked on paper.

### Conflict of Interest Statement

The authors declare that the research was conducted in the absence of any commercial or financial relationships that could be construed as a potential conflict of interest.

## References

[1] Kumar S Yadav N Pandey S Thelma BK. Advances in the discovery of genetic risk factors for complex forms of neurodegenerative disorders: contemporary approaches, success, challenges, and prospects. J Genet. (2018) 97:625–48. 10.1007/s12041-018-0953-5 30027900

[2] van Den Eden SK Tanner CM Bernstein AL Fross RD Leimpeter A Bloch DA Incidence of Parkinson's disease: variations by age, gender and race ethnicity. Am J Epidemol. (2003) 157:1015–22. 10.1093/aje/kwg068 12777365

[3] Wang M-D Little J Gomes J Cashman NR Krewski D. Identification of risk factors associated with onset and progression of amyotrophic lateral sclerosis using systematic review and meta-analysis. NeuroToxicol. (2017) 61:101–30. 10.1016/j.neuro.2016.06.015 27377857

[4] Martin LJ Mitochondrial and cell death mechanisms in neurodegenerative disease. Pharmaceuticals. (2010) 3:839–915. 10.3390/ph3040839 21258649 PMC3023298

[5] Polymeropoulos MH Lavedan C Leroy E Ide SE Dehejia A Dutra A. Mutation in the α-synuclein gene identified in families with Parkinson's disease. Science. (1997) 276:2045–7. 10.1126/science.276.5321.2045 9197268

[6] Singleton AB Farrer M Johnson J Singleton A Hague S Kachergus J. Alpha-synuclein locus triplication causes Parkinson's disease. Science. (2003) 302:841. 10.1126/science.1090278 14593171

[7] Roozenbeek B Maas Al Menon DK. Changing patterns in the epidemiology of traumatic brain injury. Nat Rev Neurol. (2013) 9:231–6. 10.1038/nrneurol.2013.22 23443846

[8] Mendez MF Paholpak P Lin A Zhang JY Teng E. Prevalence of traumatic brain injury in early versus late-onset Alzheimer's disease. J Alzheimers Dis. (2015) 47:985–93. 10.3233/JAD-143207 26401777 PMC4753056

[9] Crane PK Gibbons LE Dams-O'Connor K Trittschuh E Leverenz JB Keene CD Association of traumatic brain injury with late-life neurodegenerative conditions and neuropathological findings. JAMA Neurol. (2016) 73:1062–9. 10.1001/jamaneurol.2016.1948 27400367 PMC5319642

[10] Cruz-Haces M Tang J Acosta G Fernandez J Shi R. Pathological correlations between traumatic brain injury and chronic neurodegenerative diseases. Transl Neurodegener. (2017) 6:20. 10.1186/s40035-017-0088-2 28702179 PMC5504572

[11] Hubble JP Cao T Hassanein RE Neuberger JS Koller WC. Risk factors for Parkinson's disease. Neurology. (1993) 43:1693–97. 10.1212/WNL.43.9.1693 8414014

[12] McKee AC Gavett BE Stern RA Nowinski CJ Cantu RC Kowall NW. TDP-43 proteinopathy and motor neuron disease in chronic traumatic encephalopathy. J Neuropath Exp Neurol. (2010) 69:918–29. 10.1097/NEN.0b013e3181ee7d85 20720505 PMC2951281

[13] Beretta S Carri MT Beghi E Chio A Ferrarese C. The sinister side of Italian soccer. Lancet Neurol. (2003) 2:656–7. 10.1016/S1474-4422(03)00579-9 14572730

[14] Chio A Benzi G Dossena M Mutani R Mora G. Severely increased risk of amyotrophic lateral sclerosis among Italian professional football players. Brain. (2005) 128:472–6. 10.1093/brain/awh373 15634730

[15] Scarmeas N Shih T Stern Y Ottman R Rowland LP. Premorbid weight, body mass, and varsity athletics in ALS. Neurology. (2002) 59:773–5. 10.1212/WNL.59.5.773 12221178

[16] Beghi E Logroscino G Chio A Hardiman O Millul A Mitchell D Amyotrophic lateral sclerosis, physical exercise, trauma and sports: result of a population-based pilot case-control study. Amyotroph Lateral Scler. (2010) 11:289–92. 10.3109/17482960903384283 20433412 PMC3513269

[17] Chen H Richard M Sandler DP Umbach DM Kamel F. Head injury and amyotrophic lateral sclerosis. Am J Epidemiol. (2007) 166:810–6. 10.1093/aje/kwm153 17641152 PMC2239342

[18] Brettschneider J Del Tredici K Lee VM Trojanowski JQ. Spreading of pathology in neurodegenerative diseases: a focus on human studies. Nat Rev Neurosci. (2015) 16:109–20. 10.1038/nrn3887 25588378 PMC4312418

[19] Brambrink AM Ichord RN Martin LJ Koehler RC Traystman RJ. Poor outcome after hypoxia-ischemia in newborns is associated with physiological abnormalities during early recovery. Exp Toxic Pathol. (1999) 51:151–62. 10.1016/S0940-2993(99)80089-X 10192584

[20] Treble-Barna A Wade Sl Martin LJ Pilipenko V Yeates KO Taylor HG. Influence of dopamine-related genes on neurobehavioral recovery after traumatic brain injury during early childhood. J Neurotrauma. (2017) 34:1919–31. 10.1089/neu.2016.4840 28323555 PMC5455258

[21] Esih K Goricar K Dolzan V Rener-Primec Z. The association between antioxidant enzyme polymorphisms and cerebral palsy after perinatal hypoxic-ischaemic encephalopathy. Eur J Paed Neurol. (2016) 20:704–8. 10.1016/j.ejpn.2016.05.018 27302388

[22] Bi D Chen M Zhang X Wang H Xia L Shang Q. The association between sex-related interleukin-6 gene polymorphisms and the risk for cerebral palsy. J Neuroflamm. (2014) 11:100. 10.1186/1742-2094-11-100 24903966 PMC4060844

[23] Calkavur S Akisu M Olukman O Balim Z Berdeli A Cakmak B. Genetic factors that influence short-term neurodevelopmental outcome in term hypoxic-ischaemic encephalopathic neonates. J Intl Med Res. (2011) 39:1744–56. 10.1177/147323001103900517 22117975

[24] Wu YW Croen LA Torres AR De Water JV Grether JK Hsu NN. Interleukin-6 genotype and risk for cerebral palsy in term and near-term infants. Ann Neurol. (2009) 66:663–70. 10.1002/ana.21766 19938160

[25] Rajatileka S Odd D Robinson MT Spittle AC Dwomoh L Williams M. Variants of the EAAT2 glutamate transporter gene promoter are associated with cerebral palsy in preterm infants. Mol Neurobiol. (2018) 55:2013–24. 10.1007/s12035-017-0462-1 28271401 PMC5840247

[26] Ferriero DM. Neonatal brain injury. New Engl J Med. (2004) 351:1985–95. 10.1056/NEJMra041996 15525724

[27] Martin LJ Semenkow S Hanaford A Wong M. The mitochondrial permeability transition pore regulates Parkinson's disease development in mutant α-synuclein transgenic mice. Neurobiol Aging. (2014) 35:1132–52. 10.1016/j.neurobiolaging.2013.11.008 24325796 PMC3948207

[28] Gurney ME Pu H Chiu AY Dal Canto MC Polchow CY Alexander DD. Motor neuron degeneration in mice that express a human Cu, Zn superoxide dismutase mutation. Science. (1994) 264:1772–5. 10.1126/science.8209258 8209258

[29] Jankowsky JL Slunt HH Ratovitski T Jenkins NA Copeland NG Borchelt DR. Co-expression of multiple transgenes in mouse CNS: a comparison of strategies. Biomolec Eng. (2001) 17:157–65. 10.1016/S1389-0344(01)00067-3 11337275

[30] LaClair KD Donde A Ling JP Jeong YH Chhabra R Martin LJ. Depletion of TDP-43 decreases fibril and plaque β-amyloid and exacerbates neurodegeneration in an Alzheimer's mouse model. Acta Neuropathol. (2016) 132:859–73. 10.1007/s00401-016-1637-y 27785573 PMC5131701

[31] Martin LJ Liu Z Chen K Price AC Pan Y Swaby JA. Motor neuron degeneration in amyotrophic lateral sclerosis mutant superoxide dismutase-1 transgenic mice: mechanisms of mitochondriopathy and cell death. J Comp Neurol. (2007) 500:20–46. 10.1002/cne.21160 17099894

[32] Martin LJ Gertz B Pan Y Price AC Molkentin JD Chang Q. The mitochondrial permeability transition pore in motor neurons: involvement in the pathobiology of ALS mice. Exp Neurol. (2009) 218:333–46. 10.1016/j.expneurol.2009.02.015 19272377 PMC2710399

[33] Natale JE Cheng Y Martin LJ. Thalamic neuron apoptosis emerges rapidly after cortical damage in immature mice. Neuroscience. (2002) 112:665–76. 10.1016/S0306-4522(02)00098-2 12074908

[34] Mueller D Shamblott MJ Fox HE Gearhart JD Martin LJ. Transplanted human embryonic germ cell-derived neural stem cells replace neurons and oligodendrocytes in the forebrain of neonatal mice with excitotoxic brain damage. J Neurosci Res. (2005) 82:592–608. 10.1002/jnr.20673 16247803

[35] Martin LJ Liu Z. Adult olfactory bulb neural precursor cell grafts provide temporary protection from motor neuron degeneration, improve motor function, and extend survival in amyotrophic lateral sclerosis mice. J Neuropathol Exp Neurol. (2007) 66:1002–18. 10.1097/nen.0b013e318158822b 17984682

[36] Wong M Martin LJ. Skeletal muscle-restricted expression of human SOD1 causes motor neuron degeneration in transgenic mice. Hum Mol Genet. (2010) 19:2284–302. 10.1093/hmg/ddq106 20223753 PMC2865380

[37] Martin LJ Pan Y Price AC Sterling W Copeland NG Jenkins NA. Parkinson's disease α-synuclein transgenic mice develop neuronal mitochondrial degeneration and cell death. J Neurosci. (2006) 26:41–50. 10.1523/JNEUROSCI.4308-05.2006 16399671 PMC6381830

[38] Martin LJ Brambrink AM Price AC Kaiser A Agnew DM Ichord RN. Neuronal death in newborn striatum after hypoxia-ischemia is necrosis and evolves with oxidative stress. Neurobiol Dis. (2000) 7:169–91. 10.1006/nbdi.2000.0282 10860783

[39] Wu D Martin LJ Northington FJ Zhang J. Oscillating-gradient diffusion magnetic resonance imaging detects acute subcellular changes in the mouse forebrain after neonatal hypoxia-ischemia. J Cereb Blood Flow Metabol. (2018). 10.1177/0271678X18759859. [Epub ahead of print]. 29436246 PMC6668516

[40] Wong-Riley M. Changes in the visual system of monocularly sutured or enucleated cats demonstrable with cytochrome oxidase histochemistry. Brain Res. (1979)171:11–28. 10.1016/0006-8993(79)90728-5 223730

[41] Al-Abdulla NA Martin LJ. Apoptosis of retrogradely degenerating neurons occurs in association with the accumulation of perikaryal mitochondria and oxidative damage to the nucleus. Am J Pathol. (1998) 153:447–56. 10.1016/S0002-9440(10)65588-5 9708805 PMC1852973

[42] Martin LJ Brambrink A Koehler RC Traystman RJ. Primary sensory and forebrain motor systems in the newborn brain are preferentially damaged by hypoxia-ischemia. J Comp Neurol. (1997) 377:262–85. 10.1002/(SICI)1096-9861(19970113)377:2<262::AID-CNE8>3.0.CO;2-1 8986885

[43] Northington FJ Ferriero DM Graham EM Traystman RJ Martin LJ. Early neurodegeneration after hypoxia-ischemia in neonatal rat is necrosis while delayed neuronal death is apoptosis. Neurobiol Dis. (2001) 8:207–19. 10.1006/nbdi.2000.0371 11300718

[44] Mueller-Burke D Koehler RC Martin LJ. Rapid NMDA receptor phosphorylation and oxidative stress precede striatal neurodegeneration after hypoxic ischemia in newborn piglets and are attenuated with hypothermia. Int J Devl Neurosci. (2008) 26:67–76. 10.1016/j.ijdevneu.2007.08.015 17950559 PMC2692732

[45] Martin LJ Price AC McClendon KB Al-Abdulla NA Subramaniam JR Wong PC. Early events of target deprivation/axotomy-induced neuronal apoptosis *in vivo*: oxidative stress, DNA damage, p53 phosphorylation and subcellular redistribution of death proteins. J Neurochem. (2003) 85:234–47. 10.1046/j.1471-4159.2003.01659.x 12641745

[46] Giasson BI Duda JE Murray IVJ Chen Q Souza JM Hurtig HI. Oxidative damage linked to neurodegeneration by selective alpha-synuclein nitration in synucleinopathy lesions. Science. (2000) 290:985–9. 10.1126/science.290.5493.985 11062131

[47] Kopp VJ Jobson M. Does isoflurane or isoflurane plus hyperoxia induce apoptotic cell death? Anesth Analg. (2013) 117:1023. 10.1213/ANE.0b013e3182a231b5 24057952

[48] Martin LJ Kaiser A Price AC. Motor neuron degeneration after sciatic nerve avulsion in adult rat evolves with oxidative stress and is apoptosis. J Neurobiol. (1999) 40:185–201. 10.1002/(SICI)1097-4695(199908)40:2<185::AID-NEU5>3.0.CO;2-# 10413449

[49] Chuang PY Conley P Poloyac SM Okonkwo DO Ren D Sherwood PR. Neuroglobin genetic polymorphisms and their relationship to functional outcomes after traumatic brain injury. J Neurotrauma. (2010) 27:999–1006. 10.1089/neu.2009.1129 20345238 PMC2943497

[50] Hoh NZ Wagner AK Alexander SA Clark RB Beers SR Okonkwo DO. Bcl2 genotypes: functional and neurobehavioral outcomes after severe traumatic brain injury. J Neurotrauma. (2010) 27:1413–27. 10.1089/neu.2009.1256 20504155 PMC2967822

[51] Gallek MJ Conley YP Sherwood PR Horowitz MB Kassam A Alexander SA APOE genotype and functional outcome following aneurismal subarachnoid hemorrhage. Biol Res Nurs. (2009) 10:2050212 10.1177/1099800408323221 PMC2744368 19017669

[52] Martin LJ Chen K Liu Z. Adult motor neuron apoptosis is mediated by nitric oxide and Fas death receptor linked by DNA damage and p53 activation. J Neurosci. (2005) 25:6449–59. 10.1523/JNEUROSCI.0911-05.2005 16000635 PMC6725285

[53] Chang Q Martin LJ. Glycine receptor channels in spinal motoneurons are abnormal in a transgenic mouse model of amyotrophic lateral sclerosis. J Neurosci. (2011) 31:2815–27. 10.1523/JNEUROSCI.2475-10.2011 21414903 PMC3081715

[54] Chang Q Martin LJ. Voltage-gated calcium channels are abnormal in cultured spinal motoneurons in the G93A-SOD1 transgenic mouse model of ALS. Neurobiol Dis. (2016) 93:78–95. 10.1016/j.nbd.2016.04.009 27151771 PMC4930677

[55] Van Zundert B Izaurieta P Fritz E Alvarez FJ. Early pathogenesis in the adult-onset neurodegenerative disease amyotrophic lateral sclerosis. J Cell Biochem. (2012) 113:3301–12. 10.1002/jcb.24234 22740507 PMC3886915

[56] Bendotti C Calvaresi N Chiveri L Prelle A Moggio M Braga M. Early vacuolization and mitochondrial damage in motor neurons of FALS mice are not associated with apoptosis or with changes in cytochrome oxidase histochemical reactivity. J Neurol Sci. (2001) 191:25–33. 10.1016/S0022-510X(01)00627-X 11676989

[57] Koehler RC Yang Z-J Lee JK Martin LJ. Perinatal hypoxic-ischemic brain injury in large animal models: relevance to human neonatal encephalopathy. J Cereb Blood Flow Metabol. (2018) 38:2092–11. 10.1177/0271678X18797328 30149778 PMC6282216

[58] Braak H de Vos RAI Bohl J Tredici KD. Gastric α-synuclein immunoreactive inclusions in Meissner's and Auerbach's plexuses in cases staged for Parkinson's disease-related brain pathology. Neurosci Lett. (2006) 396:67–72. 10.1016/j.neulet.2005.11.012 16330147

[59] Lesuisse C Martin LJ. Long-term culture of mouse cortical neurons as a model for neuronal development, aging, and death. J Neurobiol. (2002) 51:9–23. 10.1002/neu.10037 11920724

[60] Maroteaux L Campanelli JT Scheller RH Synuclein: a neuron-specific protein localized to the nucleus and presynaptic nerve terminals. J Neurosci. (1998) 8:2804–15. 10.1523/JNEUROSCI.08-08-02804.1988 PMC6569395 3411354

[61] Nakamura K Nemani VM Wallender EK Kaehlcke K Ott M Edwards RH. Optical reporters for the conformation of α-synuclein reveal a specific interaction with mitochondria. J Neurosci. (2008) 28:12305–17. 10.1523/JNEUROSCI.3088-08.2008 19020024 PMC6671709

[62] Serpell LC Berriman J Jakes M Goedert M Crowther RA. Fiber diffraction of synthetic alpha synuclein filaments shows amyloid-like cross-beta conformation. Proc Natl Acad Sci USA. (2000) 97:4897–902. 10.1073/pnas.97.9.4897 10781096 PMC18329

[63] Conway KA Lee SJ Rochet JC Ding TT Williamson RE Lansbury PT Jr. Acceleration of oligomerization, not fibrilization, is a shared property of both alpha-synuclein mutations linked to early-onset Parkinson's disease: implications for pathogenesis and therapy. Proc Natl Acad Sci USA. (2000) 97:571–6. 10.1073/pnas.97.2.571 10639120 PMC15371

[64] Caughey B Lansbury PT Protofibrils, pores, fibril, and neurodegeneration: separating the responsible protein aggregates from the innocent bystanders. Annu Rev Neurosci. (2003) 26:267–98. 10.1146/annurev.neuro.26.010302.081142 12704221

[65] Ischiropoulos H Oxidative modification of alpha-synuclein. Ann NY Acad Sci. (2003) 991:93–100. 10.1111/j.1749-6632.2003.tb07466.x 12846977

[66] Hsu LJ Sagara Y Arroyo A Rockenstein E Sisk A Mallory M. Alpha-synuclein promotes mitochondrial deficit and oxidative stress. Am J Pathol. (2000) 157:401–10. 10.1016/S0002-9440(10)64553-1 10934145 PMC1850140

[67] Junn E Mouradian MM. Human alpha-synuclein over-expression increases intracellular reactive oxygen species levels and susceptibility to dopamine. Neurosci Lett. (2002) 320:146–50. 10.1016/S0304-3940(02)00016-2 11852183

[68] Tabrizi SJ Orth M Wilkinson JM Taanman JW Warner TT Cooper JM. Expression of mutant alpha-synuclein causes increased susceptibility to dopamine toxicity. Hum Mol Genet. (2000) 9:2683–9. 10.1093/hmg/9.18.2683 11063727

[69] Dickson DW. Building a more perfect beast: APP transgenic mice with neuronal loss. Am J Pathol. (2004) 164:1143–6. 10.1016/S0002-9440(10)63202-6 15039203 PMC1615338

[70] Perez SE Dar S Ikonomovic MD DeKosky ST Mufson EJ. Cholinergic forebrain degeneration in the APPswe/PS1ΔE9 transgenic mouse. Neurobiol Dis. (2007) 28:3–15. 10.1016/j.nbd.2007.06.015 17662610 PMC2245889

[71] Xu G Gonzales V Borchelt DR. Aβ deposition does not cause the aggregation of endogenous tau in transgenic mice. Alzh Dis Assoc Dis. (2002) 3:196–201 10.1097/00002093-200207000-00011 12218652

[72] Braak H Alafuzoff I Arzberger T Kretzschmar H Del Tredici K. Staging of Alzheimer disease-associated neurofibrillary pathology using paraffin sections and immunocytochemistry. Acta Neuropathol. (2006) 112:389–404. 10.1007/s00401-006-0127-z 16906426 PMC3906709

[73] Braak H Thal DR Ghebremedhin E Del Tredici K Stages of the pathologic process in Alzheimer's disease: age categories from 1 to 100 years. J Neuropath Exp Neurol. (2011) 70:960–9. 10.1097/NEN.0b013e318232a379 22002422

[74] Sze C-I Troncoso JC Kawas C Mouton P Price DL Martin LJ Loss of the presynaptic vesicle protein synaptophysin in hippocampus correlates with cognitive decline in Alzheimer's disease. J Neuropathol Exp Neurol. (1997) 56:933–94. 10.1097/00005072-199708000-00011 9258263

[75] Terry RD Masliah E Salmon DP Butters N Deteresa R Hill R. Physical basis of cognitive alterations in Alzheimer's disease: synapse loss is the major correlate of cognitive impairment. Ann Neurol. (1991) 30:572–80. 10.1002/ana.410300410 1789684

[76] Choudhry F Howlett DR Richardson JC Francis PT Williams RJ. Pro-oxidant diet enhances β/γ secretase-mediated APP processing in APP.PS1 transgenic mice Neurobiol Aging. (2012) 33:960–8. 10.1016/j.neurobiolaging.2010.07.008 20724034

[77] Burnsed JC Chavez-Valdez R Shanaz-Hossain M Kesevan K Martin LJ Zhang J. Hypoxia-ischemia and therapeutic hypothermia in the neonatal mouse brain- a longitudinal study. PLoS One. (2015) 10:e0118889. 10.1371/journal.pone.0118889 25774892 PMC4361713

[78] Martin LJ Chang Q. DNA damage response and repair, DNA methylation, and cell death in human neurons and experimental animal neurons are different. J Neuropath Exp Neurol. (2018) 77:636–55. 10.1093/jnen/nly040 29788379 PMC6005106

